# Segmental Rectum Resection for Deep Endometriosis and Excision Similarly Improve Sexual Function and Pain

**DOI:** 10.3390/clinpract13040071

**Published:** 2023-07-07

**Authors:** Fernanda de Almeida Asencio, Raphael Jose Palhares Fins, Carolina Kami Mitie, Anastasia Ussia, Arnauld Wattiez, Helizabet Salomao Ribeiro, Paulo Ayrosa Ribeiro, Philippe Robert Koninckx

**Affiliations:** 1 Department of Gynaecology Endoscopy, Santa Casa de São Paulo Hospital, São Paulo 01221-010, Brazil; fedealmeida1982@hotmail.com (F.d.A.A.); raphaelfins@gmail.com (R.J.P.F.); helizabetsalomao@gmail.com (H.S.R.); paulo.ayroza@gmail.com (P.A.R.); 2 Medicine College, University of Santa Casa de São Paulo, São Paulo 01224-001, Brazil; carolina_kami@hotmail.com; 3 Gemelli Hospital, Universtità Cattolica del Sacro Cuore, 00168 Rome, Italy; anastasia.ussia@gmail.com; 4 Department of Obstetrics and Gynaecology, University of Strasbourg, 67081 Strasbourg, France; arnaud.wattiez@wanadoo.fr; 5 Latifa Hospital, Dubai P.O. Box 9115, United Arab Emirates; 6 Department of Obstetrics and Gynecology, University Hospital Gasthuisberg, Catholic University Leuven, 3000 Leuven, Belgium

**Keywords:** endometriosis surgery, deep endometriosis, sexuality, FSFI, sexual functioning, EHP 30

## Abstract

Segmental rectum resections for indications other than endometriosis were reported to result in up to 40% sexual dysfunctions. We, therefore, evaluated sexual function after low bowel resection (*n* = 33) for deep endometriosis in comparison with conservative excision (*n* = 23). Sexual function was evaluated with the FSFI-19 (Female Sexuality Functioning Index) and EHP 30 (Endometriosis Health Profile). The pain was evaluated with visual analogue scales. Linear excision and bowel resections improved FSFI, EHP 30, and postoperative pain comparably. By univariate analysis, a decreased sexual function was strongly associated with pain both before (*p* < 0.0001) and after surgery (*p* = 0.0012), age (*p* = 0.05), and duration of surgery (*p* = 0.023). By multivariate analysis (proc logistic), the FSFI after surgery was predicted only by FSFI before or EHP after surgery. No differences were found between low bowel segmental resection and a more conservative excision. In conclusion, improving pain after surgery can explain the improvement in sexual function. A deleterious effect of a bowel resection on sexual function was not observed for endometriosis. Sexual function in women with endometriosis can be evaluated using a simplified questionnaire such as FSFI-6.

## 1. Introduction

Endometriosis, defined as “endometrium-like glands and stroma outside the uterine cavity” [1], is associated with pelvic pain and infertility. Deep and cystic ovarian endometriosis are associated with severe pain [2] and sexual dysfunction [3,4,5,6,7,8,9,10,11,12,13,14]. Surgical excision of deep endometriosis improves pain and quality of life [15], bowel function [16], and sexual function [17,18,19,20,21,22,23,24,25]. The improved sexual function is explained by the decrease in pain since chronic pelvic pain [26,27,28,29], especially deep dyspareunia [22,30,31,32,33,34,35,36,37,38,39,40] and even dysmenorrhoea [14], are strong inhibitors of sexual function. 

Excision of bowel endometriosis can be technically difficult and is complication-prone because of the associated adhesions, anatomical distortion, and bladder, ureter, or bowel surgery [41]. The surgery varies from conservative [42,43], discoid [44], or linear excision [45] to segmental bowel resections [46,47]. The type of intervention varies with the size and localization of the nodule, the risks of complications, local preferences and experience [48], the perceived importance of completeness considering microscopic endometriosis at a distance from the lesions [49,50,51], and the importance of removing all surrounding fibrosis [52].

The complications of segmental bowel resections for indications other than endometriosis are well-known [53]. After a sigmoid resection, complications are rare, and bowel leaks occur in less than 1% of cases. However, the incidence of bowel leaks increases to more than 10% for ultra-low rectum resections. Low rectum resections are further associated with lifelong urinary and bowel disturbances [54] in 30% of cases, while sexual problems [55] affect up to 40% of patients. These functional complications are considered consequences of impaired reservoir function [56,57] and neural damage [58], respectively. 

Bowel resections for deep endometriosis are expected to have fewer functional complications than resection for other indications, such as cancer since dissection is less extensive and the length of excision is shorter. After bowel resections for deep endometriosis, bowel dysfunction and quality of life [59,60,61], urinary function [62,63,64], and sexual function improve [21]. However, it remains unclear whether a bowel resection negatively affects sexual function. Although most reports did not find a difference between bowel resections and conservative excision, some reported that sexual function [65], orgasm problems [66], or sexual quality failed to improve to the expected extent [60]. 

The female sexual functioning index (FSFI) is the most widely used screening tool [67] to evaluate sexual function. FSFI evaluates six domains: desire, arousal, lubrication, orgasm, sexual satisfaction, and pain. However, it is unclear whether the FSFI, being developed as a screening tool for all types of sexual dysfunction, is appropriate to investigate the sexual dysfunction associated with endometriosis, pain, and nerve damage. Moreover, the pain domain of FSFI evaluates superficial and deep dyspareunia, which overlaps with pain symptoms associated with endometriosis. 

Without entering the surgical debate of bowel resection versus conservative excision, the potentially harmful effect of bowel resections on sexual function is important in the younger age group. We, therefore, evaluated sexual function after segmental bowel resection compared to the more conservative linear resection in a one-surgeon, one-centre setting. 

## 2. Materials and Methods

### 2.1. Study 

The Ethical Review Board of the hospital Santa Casa de Misericordia approved the study as nr 4.130.255. Before enrollment, the study was explained, and an informed consent form was signed. 

All women (*n* = 132) who underwent laparoscopic segmental resection (SR) or linear nodulectomy (LN) for bowel endometriosis between December 2019 and February 2021 at the Gynecological Endoscopy and Endometriosis department at Santa Casa de Misericórdia Hospital in São Paulo, Brazil were invited to participate. Only 61 women accepted, probably because women were reluctant to come to the hospital during the COVID pandemic. Also, discussing sexuality might be more sensitive in Brazil than in other parts of the world. After the removal of six women who had not been sexually active (*n* = 5), 23 women with a linear nodulectomy (LN) and 33 with a segmental resection (SR) were included.

Inclusion criteria were women of reproductive age who underwent surgery for histologically confirmed deep endometriosis of the rectum or rectosigmoid and who had been sexually active before and after surgery. Exclusion criteria included postoperative complications, a history of psychiatric disorder, or the use of psychotropic drugs. 

### 2.2. Outcome

Sexual function was evaluated with the 19-item FSFI (Female Sexual Function Index) [68], which had been translated and validated into Portuguese [69,70,71]. The FSFI evaluates separately desire, arousal, lubrification, orgasm, satisfaction, and pain on a 0 or 1 to 6 scale resulting in a range of 2 to 36. A high score indicates better sexual function, and a score below 26 indicates sexual dysfunction [72]. It is important that the pain domain evaluates both superficial and deep dyspareunia. 

The Endometriosis Health Profile Questionnaire (EHP-30) [5] consists of 30 questions scored from 0 to 4, and the sum is successively transformed into a 0 to 100 scale. Important is that a lower scale indicates better functioning and that 10 questions specifically address the impact of pain. 

Dysmenorrhea, dyspareunia, dyschezia, dysuria, and chronic pelvic pain (CPP) were evaluated by visual analogue scales (VAS), 0 to 10. 

Demographic and surgical data such as age, parity, infertility, medication, BMI, and duration of surgery were retrieved from the records. 

### 2.3. Surgery 

All surgeries were performed by PAAR and HSAAR. All women had preoperatively a transvaginal ultrasound and/or a pelvic MRI, but the surgery technique was decided during surgery. Bowel endometriosis lesions estimated to be smaller than 3 cm were removed by linear resection using a linear stapler. Larger lesions or those involving more than 50% of the bowel circumference [73] were treated by a limited segmental bowel resection. Excision was visually complete without safety margins, and care was taken not to damage the nerves using nerve-sparing principles.

After initiating the pneumoperitoneum, one 11 mm and three accessory 5 mm trocars were inserted, and the pelvic cavity was inspected for endometriosis. If necessary, an adhesiolysis and excision of cystic ovarian endometriomas with temporary suspension of the ovaries were performed. After bilateral ureterolysis, the pararectal space was opened, the hypogastric nerves were identified, and the rectovaginal space was dissected to isolate the intestinal lesion(s). Only then was it decided to perform a linear or segmental resection. A linear resection of a nodule was performed, as described [74,75,76], with a linear stapler (Endogia 30 mm, Covidien) without much dissection and thus without affecting the nerves lateral and posterior to the bowel. A segmental bowel resection required a complete mobilization of the bowel above and below the disease. Care was taken to preserve the vascular and nervous supply. The distal loop was sectioned with a linear stapler (Endo GIA tri staple 45 mm, Covidien) 1 cm below the disease. The proximal loop was exteriorized through an extended right lateral trocar incision and sectioned 1 cm above the lesion, thus generating a limited resection [77]. The end-to-end anastomosis was performed with a transanal circular stapler (DSTTM EEA series; Medtronic) and checked for leakage. 

### 2.4. Statistics

Statistical analyses were carried out with the SAS system [78], using Spearman and Pearson correlations, non-parametric Mann-Whitney or Wilcoxon tests, and multivariate analysis with logistic regression (proc logistics) or the non-parametric Anova (Proc GLM). Mean and standard deviations (SD) are indicated unless otherwise indicated. Exact *p* values are given as suggested by the American statistical association [79,80]. However, we still used the word ‘significant’, despite the definition of <0.05 being arbitrary and despite the *p*-value fallacy with frequent erroneous conclusions in medicine considering that traditional frequentist statistics can only refute, but cannot confirm a hypothesis’ independent and dependent variables. Data summation, such as sumpain, requires validation; however, if two variables are very (+++) strongly associated, they generally carry the same information. In multivariate exploratory models, either one or the other but not both variables will independently reach a *p*-value lower than 0.05. Considering the inherent variability of variables, the sum of these strongly associated variables is possibly a better estimate than each variable separately. 

## 3. Results

The 23 women who underwent a linear nodulectomy (LN) and the 33 who underwent a segmental bowel resection (SR) are comparable (Table 1). Not surprisingly, women undergoing a segmental resection experienced slightly more pain and exhibited larger nodules, and the affected bowel segment was longer. 

After surgery, all parameters such as desire, arousal, lubrification, orgasm, pain, the FSFI score, the EHP 30 score, dysmenorrhea, dyspareunia, dyschezia, dysuria, and chronic pelvic pain (Table 2) improved significantly, whether performed by linear excision or segmental bowel resection. By two-way analysis of variance, none of these parameters showed a significant difference in improvement between segmental bowel resection or linear excision. To increase the power of the analysis, we subsequently reduced the variables by grouping those carrying similar information. 

**Figure 1:**
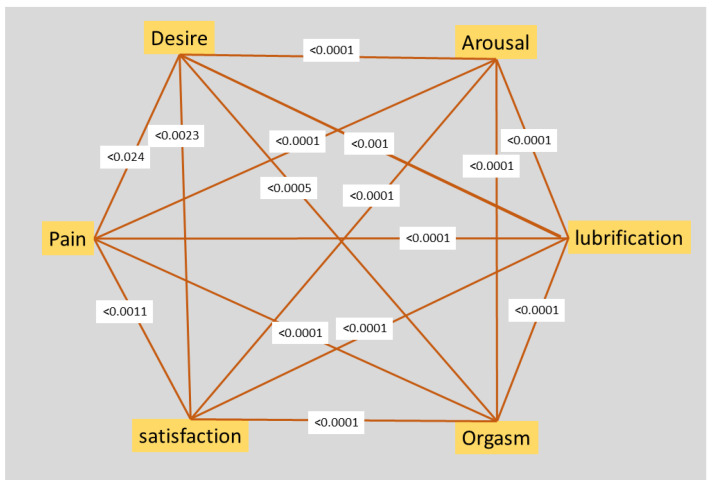
Spearman Correlations of the 6 FSFI domains before surgery. *p* values are indicated.

The six domains of the FSFI strongly correlated with each other before (Figure 1) and after (not shown since similar) surgery. Also, EHP30 scores correlated negatively (since low scores have a negative valence in FSFI, while positive in EHP30) with all FSFI domains as desire (*p* = 0.024), arousal, lubrification, orgasm, and satisfaction before surgery (all *p* < 0.0001) and after surgery (all domains *p* < 0.0001). Therefore, we only used FSFI and EHP-30 as independent variables for further analysis.

**Figure 2:**
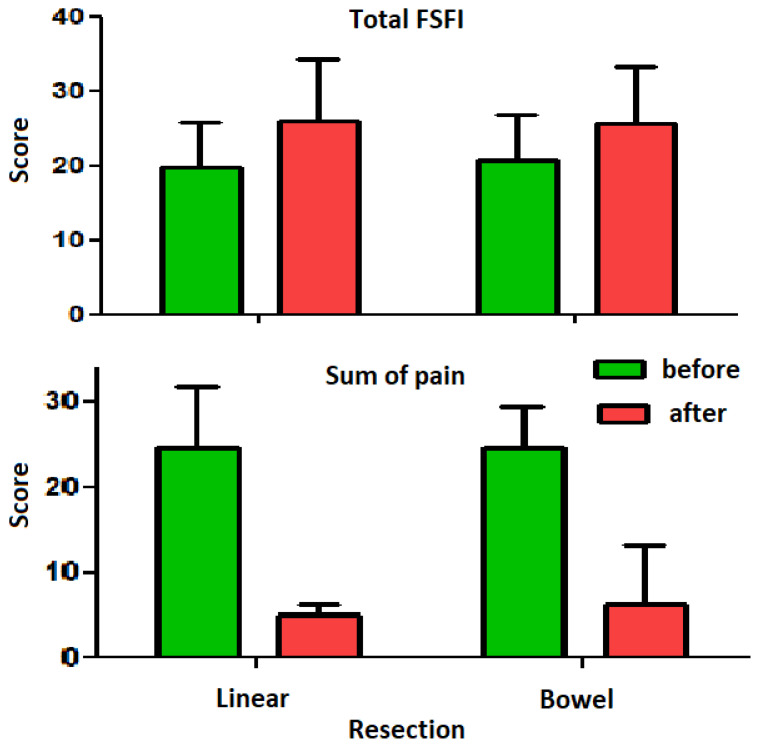
Surgery, whether performed by linear excision or bowel resection, improves endometriosis-associated pain (*p* < 0.0001) and sexual function (*p* < 0.0001).

Surgery, whether performed by linear excision or bowel resection, improves endometriosis-associated pain (*p* < 0.0001) and sexual function (*p* < 0.0001) (Figure 2). Also, pain estimations correlated with each other. Before surgery, dysmenorrhoea correlated with dyspareunia (*p* = 0.0045) and chronic pain (*p* = 0.00011), while dyspareunia correlated with chronic pelvic pain (*p* ≤ 0.0001). After surgery, dysmenorrhoea correlated with dyschezia (*p* = 0.0371) and CPP (*p* = 0.0385), deep dyspareunia with dyschezia (*p* = 0.0016) and CPP (*p* = 0.0059), and dyschezia with CPP (*p* = 0.0006). For further analysis, deep dyspareunia was excluded since the information was similar to that of the pain domain (deep and superficial dyspareunia) of FSFI before and after surgery (*p* < 0.0001). Dysmenorrhea, dysuria, and CPP were grouped as SumPain since they strongly correlated with FSFI (all *p* < 0.0001). 

Considering the strong correlation of FSFI (*p* = 0.0166) and EHP30 (*p* = 0.0009) before and after surgery, a specific effect of bowel resections on sexuality after surgery needed to be corrected by FSFI and EHP-30 before surgery. In addition, EHP-30, Sumpain, and dyschezia after surgery and other variables such as age and duration of surgery and volume of nodules had to be used as co-variables. By univariate analysis, the FSFI after surgery correlated positively with FSFI before surgery (*p* = 0.0131), EHP-30 after surgery (*p* < 0.0001), and improvement of EFP-30 (*p* = 0.0002) with the duration of surgery (*p* = 0.0235) and the presence of a second nodule (*p* < 0.0001), and correlated negatively with a hysterectomy (*p* = 0.0056) and age (*p* = 0.0448). By multivariate analysis (proc logistics), the only predictor of FSFI after surgery was the EHP30 after surgery (*p* < 0.0001) or the FSFI before surgery (*p* < 0.0001), without a significant additive effect of the type of surgery of any other variable.

## 4. Discussion

These data confirm the efficacy of surgery for deep endometriosis in reducing pelvic pain and improving sexual function (FSFI-19) and quality of life (EHP-30) [15,16,17,18,19,20,21,22,23,24,25]. Restricted segmental bowel resections and linear excisions have a comparable effect on sexual function, suggesting that the decreased sexual function after segmental rectum resections for diseases other than endometriosis (mostly cancers) cannot be extrapolated to endometriosis. However, the conclusion that a (restricted) bowel resection for endometriosis does not affect sexual function more than a conservative excision should be considered cautiously. Our series is small, and the interpretation requires a full understanding of independent and dependent variables as well as the content, similarities, and differences of FSFI, EHP, and pain scales in gynaecology. Dyspareunia represents 1/6th of FSFI; impaired functioning because of pain is 1/3rd of EHP-30; clinical gynaecological pain consists of severity, radiation, centralization, and cross-over of dysmenorrhoea, CPP, dyspareunia, dyschezia, and dysuria. Considering the many and partially overlapping variables, it cannot be excluded that a very large trial considering all parameters, including the severity of the nodule [81], might find a specific effect of bowel resections. However, eventual specific effects of bowel resection on sexuality are not expected to be clinically important. Considering the variability in endometriosis surgery and knowing that sexual function is reduced after posterolateral parametrial excision [82], a randomized controlled trial stratified for all variables, including the surgeon, will need to be prohibitively large and probably not ethical to perform [48]. This emphasizes the importance of short bowel resections with limited dissection and nerve-sparing for women with bowel endometriosis

The 19-item FSFI is the widely used tool to evaluate the different aspects of sexual functioning. However, in women with endometriosis, sexual function is mainly affected by pain and less so by other neurological and psychological factors such as mental health, feelings of femininity, and relationships [83,84,85]. This explains the strong correlations between the six domains of the FSFI. Therefore, the six domains of the 19-item FSFI cannot be considered independent variables in women with endometriosis, and the reduced six-item FSFI score can probably replace the FSFI-19.

Pain is a strong factor in decreasing sexual function. Therefore, the FSFI evaluates dyspareunia while the EHP 30 assesses the effect of pain. In gynaecology, pain is estimated by the severity and radiation of symptoms such as dyspareunia, dysmenorrhoea, CPP, and dyschezia, which are intercorrelated because of the underlying pathology. Sexual functioning is influenced by, besides pain, psychological factors such as fear of pain decreasing arousal, lubrication, and vaginal entry restriction [10,22,86]. However, in women with endometriosis, the relative importance of pain and other factors, as evaluated in FSFI or EHP-30, remains unclear. The same holds for the decreased sexual function with age and with a longer duration of surgery or the severity of the disease. Understanding the association of all types of pelvic pain and its cerebralisation [87] renders it difficult to interpret reported data when only specific types of pelvic pain are evaluated. Often deep dyspareunia is emphasized as a potent inhibitor of the sexual response and a modifier of behaviour, with women developing Hypoactive Sexual Desire Disorder (HSDD) or arousal disorder [3,22,30,31,32,33,34,35,36,37,38,39]. Dyspareunia doubles the risk of sexual desire disorders, with dysmenorrhea tripling said risk and chronic pelvic pain triples the probability of disorders of sexual satisfaction and orgasm. This explains why women with and without endometriosis had an FSFI below 26 in 51% and 17.5% [34], respectively. It also explains the wide range of sexual problems experienced by 32–73% of women with endometriosis [6,7,8,9,12,54], which might explain persisting sexual dysfunction after surgery caused by factors other than pain.

Our data confirm that surgery reduces pelvic pain [15,17,18,19,20,21,22] and improve sexual functioning. Dyspareunia and sexual pleasure and habit improved, as demonstrated by the Sexual Active Questionnaire taken by 135 women, with a follow-up of 2–5 years [88]. Also, surgery in women with severe dyspareunia improved the quality of sexual life, i.e., an increase in the frequency of sexual intercourse, with more satisfying orgasms and reduced difficulty relaxing during intercourse as reported by 68 women, with a follow-up of six and 12 months [19].

## 5. Conclusions

These data confirm that surgery for deep endometriosis improves the endometriosis health profile while decreasing pelvic pain and sexual dysfunction, probably as a consequence of the reduced pain. Although the data suggest that segmental rectum resection for endometriosis does not impair sexual function more than linear excision, the surgical wisdom of not doing excessive surgery remains fully valid. The FSFI, developed and validated to differentiate the different types of sexual dysfunction, might not be appropriate for evaluating sexual function in women with endometriosis since the six domains of FSFI cannot be considered independent variables. To study pain and sexuality in women affected by endometriosis, a more simplified questionnaire such as the FSFI-6 supplemented with a scale for dyspareunia, dysmenorrhoea, CPP dysuria, and dyschezia might be useful.

## Figures and Tables

**Table 1:** Characteristics of women undergoing a linear or segmental bowel resection for deep endometriosis.

	Linear Nodulectomy *n* = 23	Segmental Resection *n* = 33	*p* Value
Age	28.9 ± 6.2	37.4 ± 5.6	NS
Height (cm)	162.2 ± 5.5	163.0 ± 7.0	NS
Weight (kg)	74.3 ± 12.4	71.8 ± 13.8	NS
Para (deliveries)	1.4 ± 1.5	1.0 ± 1.2	NS
Deep endo volume (mL)	1.3 ± 1.3	6.2 ± 6.9	0.0006
Duration of surgery (min)	186 ± 50	204 ± 61	NS
Length of resection (cm)	1.4 ± 1.3	9.7 ± 3.6	<0.0001
Pain score	19.5 ± 7.9	18.1 ± 8.1	NS

**Table 2:** FSFI domains and pain symptoms (dysmenorrhea, dyspareunia, dyschezia, dysuria, and CPP) 12 months after deep endometriosis resection by linear resection of segmental bowel resection.

	Linear Resection			Segmental Resection		
	Before	After	*p* Value	Before	After	*p* Value
Desire	3.1 + 1.1	3.8 + 1.6	0.045	2.9 + 1.0	3.7 + 1.2	0.004
Arousal	3.0 + 1.1	3.8 + 1.6	0.004	3.1 + 1.0	3.9 + 1.4	0.004
Lubrification	3.8 + 1.3	4.7 + 1.7	0.022	4.1 + 1.4	4.5 + 1.6	NS
Orgasm	3.5 + 1.3	4.3 + 1.6	0.034	3.8 + 1.5	4.5 + 1.5	0.021
Satisfaction	3.8 + 1.3	4.5 + 1.5	NS	3.8 + 1.3	4.3 + 1.7	NS
Pain	2.6 + 1.6	4.6 + 1.7	0.001	2.9 + 1.6	4.6 + 1.5	<0.001
Tota FSFI	19.8 + 6.0	25.9 + 8.4	<0.001	20.8 + 5.9	25.6 + 7.7	<0.001
EHP-30	64.6 + 28.1	34.8 + 33.8	0.003	57.6 + 31.2	35.3 + 32.5	<0.001
Dysmenorrhoea	9.2 + 1.1	1.3 + 2.4	<0.001	8.5 + 2.5	6.9 + 3.4	<0.001
Dyspareunea	7.0 + 2.9	2.0 + 2.8	<0.001	7.1 + 3.3	4.9 + 3.8	<0.001
Dyschesia	5.2 + 3.4	1.4 + 2.8	0.005	5.3 + 4.0	3.5 + 4.5	0.001
Dysuria	1.6 + 2.7	0.3 + 0.7	0.021	1.7 ± 3.5	1.4 + 3.1	0.017
CPP	6.5 + 3.6	1.3 + 2.4	<0.001	6.9 ± 3.7	4.8 + 3.9	<0.001
The sum of all pain	24.5 + 7.2	4.9 + 1.3	<0.001	24.5 ± 7.2	6.2 + 6.9	<0.001

## Data Availability

Original data are available by request to the corresponding author.
